# A Multi-Scale Tool Orientation Generation Method for Freeform Surface Machining with Bull-Nose Tool

**DOI:** 10.3390/mi14061199

**Published:** 2023-06-05

**Authors:** Jieshi Dong, Jinming He, Song Liu, Neng Wan, Zhiyong Chang

**Affiliations:** 1 Department of Mechanical Engineering, Northwestern Polytechnical University, Xi’an 710072, China; dongjieshi@mail.nwpu.edu.cn (J.D.); hjm@mail.nwpu.edu.cn (J.H.); 2021261562@mail.nwpu.edu.cn (S.L.); wanneng@nwpu.edu.cn (N.W.); 2 Institute for Aero-Engine Smart Assembly of Shaanxi Province, Xi’an 710072, China

**Keywords:** five-axis CNC machining, multi-scale, tool orientation, bull-nose tool

## Abstract

Free-form surface parts are widely used in industries, and they consist of intricate 3D surfaces such as molds, impellers, and turbine blades that possess complex geometrical contours and demand high precision. Proper tool orientation is crucial for ensuring the efficiency and accuracy of five-axis computer numerical control (CNC) machining. Multi-scale methods have received much attention and have been widely used in various fields. They have been proven to be instrumental and can obtain fruitful outcomes. Ongoing research on multi-scale tool orientation generation methods, which aim to acquire tool orientations that satisfy both macro- and micro-scale requirements, is significantly important for improving the machining quality of workpiece surfaces. This paper proposes a multi-scale tool orientation generation method that considers both the machining strip width and roughness scales. This method also ensures a smooth tool orientation and avoids interference in the machining process. First, the correlation between the tool orientation and rotational axis is analyzed, and feasible area calculation and tool orientation adjustment methods are introduced. Then, the paper introduces the calculation method for machining strip widths on the macro-scale and the roughness calculation method on the micro-scale. Besides, tool orientation adjustment methods for both scales are proposed. Next, a multi-scale tool orientation generation method is developed to generate tool orientations that meet the macro- and micro-scale requirements. Finally, to verify the effectiveness of the proposed multi-scale tool orientation generation method, it is applied to the machining of a free-form surface. Experimental verification results have shown that the tool orientation generated by the proposed method can obtain the expected machining strip width and roughness, meeting both macro- and micro-scale requirements. Therefore, this method has significant potential for engineering applications.

## 1. Introduction

Free-form surface parts are widely used in the industry, and they usually consist of complex three-dimensional surfaces, such as turbine blades, impellers, and molds [1]. These parts have complex geometric shapes and high-precision requirements. Traditional machining methods make it difficult to meet their processing needs. Compared with three-axis computer numerical control (CNC) machine tools, five-axis CNC machine tools add two rotational axes based on the three translational axes so that the cutting tool can contact the part in any direction in the machining process [2]. Meanwhile, five-axis CNC machining has the properties of high efficiency, excellent flexibility, high precision, and good reliability; therefore, it has been widely used in free-form surface processing [3]. To better exploit the characteristics of five-axis machine tools, tool orientation is one of the critical elements in tool path planning.

The bull-nose tool, characterized by its variable diameter, circumvents the issue of zero cutting speed. In recent years, numerous researchers have conducted mechanistic modeling studies on the machining process involving bull-nose tools. During the machining process, cutting forces can induce tool deflection, which may ultimately result in unacceptable dimensional errors [4]. Budak and Altintas [5,6] developed a model to address the chatter stability issue during end milling by considering the dynamic interaction between the tool and workpiece. They represented both the tool and workpiece as multi-degree-of-freedom structures and accounted for the variation of dynamics in the axial direction. In mechanistic force models, the cutting coefficient of the tool depends on the cutting speed and tool geometry. Campa et al. [7] developed a stability model for milling compliant systems in the tool axis direction using bull-nose end mills by incorporating the analysis of modal parameters of thin floors. This model aids in selecting stable spindle speeds. Bull-nose end mills are essential for machining complex parts. Campa et al. [8] developed a three-dimensional dynamic model specifically for bull-nose tool machining of low-rigidity components. This model was applied to predict chatter vibrations during the finishing process of aeronautical parts. Subsequently, a method was introduced for chatter avoidance in the milling of flexible, thin floors using bull-nose tools. This approach is also applicable to the milling of thin walls and the turning of thin components [9]. Zhou et al. [10] investigated the impact of the lead angle on the machining process, taking into account the mechanical and dynamic variations arising from the complex geometry of the tool and workpiece. They developed an analytical model for predicting chatter stability in bull-nose end milling. Sokolov and Zhidyaev [11] employed stability analysis to identify stable cutting conditions, taking into account the elastoplastic properties of the tool and holder materials. They determined the holder-tool frequency response function using finite element modeling and applied this approach to bull-nose carbide end mills for titanium alloy. Moreover, cutting forces during machining can cause tool deformation, which subsequently affects the surface finish and dimensions of machined parts [12], leading to a reduction in the machine tool’s machining accuracy [13]. Tool deformation is highly significant for designing tool geometry and determining optimal cutting conditions [14], as well as being crucial for the part’s forming quality [15]. Given that this paper focuses on finishing machining with small machining parameters, tool deformation during machining can be considered negligible.

Multi-scale methods have been widely used in various fields. For example, they have been used in metal materials to study the surface behavior of martensitic tool steels [16] and in geometric modeling to establish geometrical simulation models [17]. In the research on surface topography, multi-scale methods have been used to analyze the surface morphology of workpieces and to realize more precise machining control and surface optimization [18,19]. In terms of monitoring the machining process, multi-scale methods have been employed to detect the state of the cutting tool in the machining process in real time [20,21]. Additionally, multi-scale methods have been used for the mechanical characterization of materials and structural design optimization [22]. Though multi-scale methods play an essential role in various fields and have achieved good results, they are generally lacking in the application of tool orientation planning compared with other methods. Therefore, studying the multi-scale tool orientation generation method to obtain tool orientations that meet both macro- and micro-scale requirements is of great significance for improving the machining quality of workpiece surfaces.

The objectives of conventional tool orientation planning mainly include avoiding interference [23], controlling machining strip widths [24], controlling roughness [25], reducing or avoiding drastic changes in tool orientation velocity [26] and acceleration [27], avoiding singularities [28], and controlling cutting forces [29]. Among these objectives, machining strip widths and roughness belong to geometric features, where the former is a macro-scale feature and the latter is a micro-scale feature [30]. This paper proposes a multi-scale tool orientation generation method that considers machining strip widths and roughness. The method also ensures no interference or smoothing of the tool orientation in the machining process.

The tool orientation significantly affects the machining strip widths, so many scholars have conducted related research. Frad et al. [31] investigated the influence of tool orientation on the machining strip widths in flat end milling and proposed a method that maximizes the machining strip widths. Wang et al. [32] developed a method for calculating a barrel cutter’s global spatial error and established the machining strip width calculation method. Additionally, they built a method that aims to obtain the maximum machining strip widths. Zhu et al. [33] selected the tool orientation for 3 + 2 axis free surface machining to improve machining efficiency by maximizing the average machining strip widths. Considering the interference problem, Fan et al. [34] provided a tool orientation planning method for maximizing the machining width by using the quadratic surface local approximation method. He et al. [35] studied the influence of the tilt angle and lifted value on the machining strip width without changing the yaw angle. According to the original tool path, they adjusted the tool orientation at each CC point to obtain a smooth tool orientation and approximate constant machining strip width. Liu et al. [36] investigated the effective cutting surface of a flat-end cutter under different tool orientations and obtained the machining strip widths at the CC point. Moreover, they proposed a tool path generation method that applies tensor metrics to the machining strip width. Hu et al. [37] developed a tool orientation optimization method that considers both the machining strip width and the machine motion capability to maximize the material removal rate. He et al. [38] provided a method of reducing the fluctuation of the machining strip widths by adjusting the tool orientation, thus making the machining edge gradually converge to the effective machined area.

The roughness significantly affects the workpiece’s performance. Therefore, the effect of the tool orientation on the roughness of the workpiece has always been a research focus, and many related studies have been conducted. Duan et al. [39] investigated the relationship between the tool orientation and surface roughness when processing 300 M steel material through experiments and developed a strategy to optimize the tool orientation. Yao et al. [40,41] studied the effect of the tool orientation on surface roughness through experiments taking TC17 titanium alloy as the research object, and they found that the surface roughness of the workpiece was better within the current inclination angle range of 30–60°. Fard et al. [42] investigated the effect of tool orientation on roughness through experiments in micro-milling. It was found that the tool’s inclination angle had a more significant impact on surface roughness. When the inclination angle was 15°, the surface roughness could be reduced by 35% compared to the absence of an inclination angle. Taking a ball-end tool as the research object, Pesice et al. [43] investigated the effect of the lead angle on roughness. They found that the roughness of the workpiece decreased as the lead angle increased (0–30°). Gdula [44] conducted experiments on the five-axis milling of blade parts using a bull-nose tool to study the effect of the tool orientation on surface roughness and morphology. It was discovered that within the range of 7–23°, the surface roughness gradually decreased as the inclination angle increased. Urbikain et al. [45] established a geometric model for oval-form cutter side milling and studied the effect of tool orientation on roughness and surface morphology. Simulation results indicated that roughness remained unchanged for different tilt angles. Koprowski et al. [46] conducted experimental research on the effect of tool orientation on surface roughness during grinding. It was found that the tilt and lead angles directly affected the structure angle, which further affected the workpiece’s surface roughness. Gao et al. [47] explored the effect of the inclination angle on surface roughness in micro ball-end milling. The experimental results demonstrated that when the inclination angle was between 0 and 45°, the surface roughness decreased as the absolute value of the inclination angle increased. Bouzakis et al. [48] designed a simulation algorithm for ball-end tool machining and studied the effect of the inclination angle on roughness. When the inclination angle was between 0 and 60°, the roughness decreased first and then increased as the angle increased. Zhang et al. [49] established a simulation model for ball-end tool milling to study the relationship between the inclination angle and roughness. The experimental results indicated that when the inclination angle increased from 0 to 30°, the roughness decreased first and then increased. Especially, when the inclination angle was 0°, the roughness was the largest. Runout, a form of inaccuracy in rotating mechanical systems, occurs when the tool deviates from its precise rotational axis during operation. This phenomenon directly impacts the surface roughness of the workpiece. Arizmendi et al. [50] developed a surface topography prediction model for end mill peripheral milling that accounts for the impact of tool vibration during machining. This model facilitates the estimation of topography, roughness values, and form errors. Arizmendi et al. [51] developed a predictive model for surface topography in ball-end milling, taking tool runout into consideration. The model assumes that runout results from a parallel offset between the tool and spindle axes. According to the findings, when the offset is minimal, the cusps, valleys between cusps, and scallops along the feed direction exhibit uniformity in shape. However, as the offset increases, the uniformity of cusps, valleys, and scallops diminishes.

In five-axis tool path planning, achieving interference-free and smooth tool orientations is also a key issue. He et al. [23] analyzed the causes of interference in the machining of convex surfaces with a bull-nose tool and developed an interpolation-based interference elimination method. Based on geometric analysis. Zhao et al. [25] provided a method for identifying interference-free tool orientations to determine the interference-free range. Ma et al. [52] proposed a method for calculating the minimum bounding rectangle of the tool projection section. They determined whether the tool orientation was within the interference-free feasible range by determining whether the points were inside the bounding rectangle. Wang et al. [53] investigated the geometric relationship between the tool and the surface of the blisk in five-axis milling and obtained the interference-free area of the tool in the machining process. Sun et al. [26] converted the tool orientation at the CC point into the rotational axis representation in the machine coordinate system. To reduce the angular velocity and acceleration of the rotational axis, they directly optimize the coordinates of the rotational axis. This improved the kinematic and dynamic performance of the five-axis CNC machine tool in machining and prevented drastic changes in the tool orientation at adjacent CC points. Lu et al. [54] developed a smooth tool path planning method for flat-end tool side milling that considered both geometric deviations and tangential constraints. The optimization algorithm could minimize the angular acceleration of the rotational axis. Fountas et al. [55] proposed a tool path global optimization method that uses intelligent algorithms to minimize machining errors, tool orientation smoothness, and production efficiency. Lu et al. [56] provided a five-axis side milling tool path generation method to smooth the rotational axis for impeller parts. This method improved tool path smoothness by directly optimizing the rotational axis and the tool reference point. Xu et al. [57] directly controlled the rotational angle in the machine coordinate system to achieve smooth changes in the tool orientation, thereby effectively alleviating tool orientation fluctuations. Additionally, based on the minimum deflection cutting force, López et al. [58] introduced a novel approach for selecting toolpaths in complex surface milling that minimizes dimensional errors caused by tool deflection, thereby enhancing the milling surface accuracy. Zha et al. [59] introduced an “evolution” method aimed at enhancing the accuracy of curved surfaces. This approach involves adjusting the part through toolpath compensation while maintaining other process variables constant, taking into account the measured deviation of the workpiece. Consequently, the method yields a product with the fewest number of iterations.

Previous research on the impact of tool orientations on machining strip width and roughness has obtained significant results. However, there has been limited investigation of tool orientation generation methods from a multi-scale perspective, and how to simultaneously satisfy both machining strip width and surface roughness at these scales through tool orientation planning is less studied. In response, this paper proposes a multi-scale tool orientation generation method that considers both machining strip width and roughness scales. The proposed method can also ensure no interference in the machining and guarantee the smoothness of the tool orientation.

The rest of this paper is organized below. In Section 2, the relationship between the tool orientation and rotational axis is analyzed, and the feasible area calculation and the tool orientation adjustment method are introduced. In Section 3, machining strip width calculation and tool orientation adjustment on the macroscale are introduced. In Section 4, roughness calculation and tool orientation adjustment on the microscale are introduced. Section 5 proposes a multi-scale tool orientation generation method that satisfies both macro-scale and micro-scale requirements. Meanwhile, it has interference-free and smooth rotational axis characteristics. In Section 6, the multi-scale method was verified experimentally with a free-form surface. Conclusions are presented in Section 7. The proposed method was validated in machining experiments on the free-form surface. The results indicated that it can effectively control the roughness and machining strip widths with no interference and a smooth rotational axis.

## 2. Preliminaries

**Figure 1:**
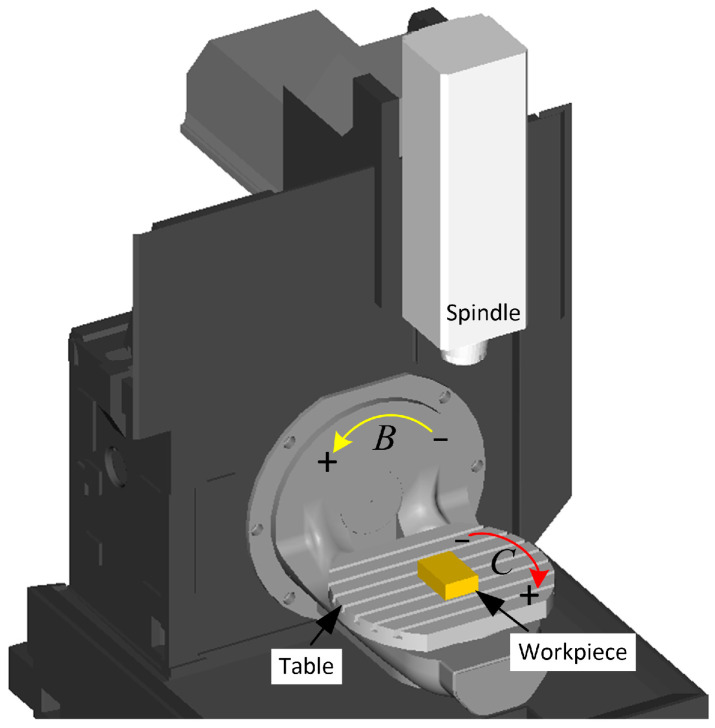
The structure of a 5-axis machine tool with a BC double turntable structure.

This paper introduces the tool orientation generation method using a 5-axis CNC machine tool with a BC double turntable structure. The machine contains three translational axes (X, Y, and Z) and two rotational axes (B and C), as shown in Figure 1. This section briefly describes two preliminaries, i.e., the relationship between tool orientation and rotational axis, and the calculation of interference-free area.

### 2.1. Analysis of the Relationship between the Tool Orientation and the Rotational Axis

**Figure 2:**
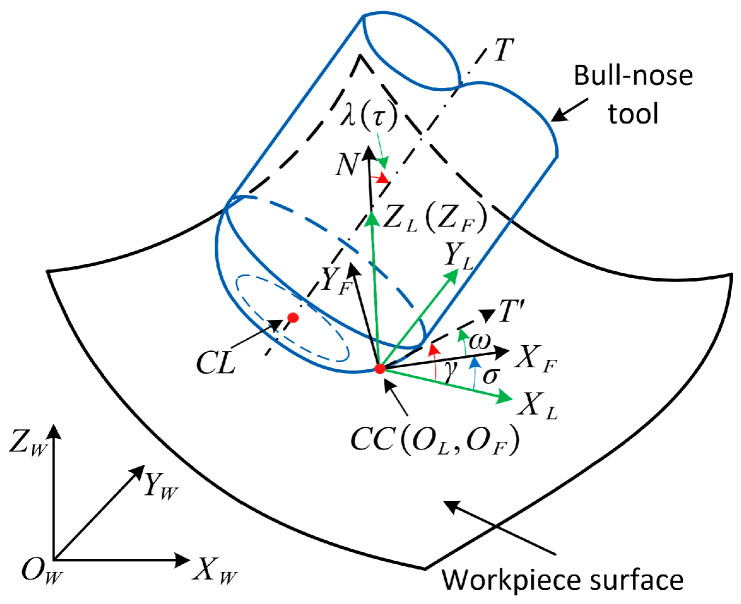
Coordinate systems in machining at the CC point.

The spatial position of the tool is determined by the cutter location data (CL Data), which contains the cutting tool location (CL) and the tool orientation (T). As illustrated in Figure 2, when the bull-nose tool is machining the CC point on the surface, the tool and the surface are tangent to each other. N represents the normal vector at the CC point, and the vector $T^{\prime }$ is the projection of T on the common tangent plane.

The surface to be machined is described in the workpiece coordinate system ($CS_{W}$). The $CS_{W}$ is defined at the time of designing the workpiece, and the geometric features of the workpiece and CC paths are described under the $CS_{W}$.

The local coordinate system ($CS_{L}$) is established at the CC point, where the origin $O_{L}$ is the CC point, $Z_{L}$ is parallel to N, $X_{L}$ is parallel to the principal direction $T_{\max}^{S}$, and $Y_{L}$ is parallel to the principal direction $T_{\min}^{S}$. At the CC point, the angle between T and N is referred to as the local inclination angle and is denoted as λ. The angle between the $T^{\prime }$ and $X_{L}$ is referred to as the local tilt angle and is denoted γ.

The feed coordinate system ($CS_{F}$) is related to the feed direction. The origin $O_{F}$ is the CC point, $Z_{F}$ is parallel to N, $X_{L}$ is parallel to the feed direction, and $Y_{L}$ is perpendicular to the feed direction. The angle between the T and N is referred to as the inclination angle and is denoted as τ. The angle between $T^{\prime }$ and $X_{L}$ is referred to as the tilt angle and is denoted as ω.

Therefore, the tool orientation can be described in two ways: (1) local inclination angle λ and local tilt angle γ; (2) inclination τ and tilt angle ω [60]. Based on Figure 2, the following relationship can be obtained:

(1) $$\{\begin{array}{l} \lambda =\tau \\ \gamma =\omega +\sigma \end{array}$$

In $CS_{W}$, the expression for the tool orientation T is:

(2) $$T_{CS_{W}}=\left(\begin{array}{c} i\\ j\\ k \end{array}\right)=\left(\begin{array}{c} \sin\lambda \cdot \cos\gamma \cdot x_{1}+\sin\lambda \cdot \sin\gamma \cdot x_{2}+\cos\lambda \cdot x_{3}\\ \sin\lambda \cdot \cos\gamma \cdot y_{1}+\sin\lambda \cdot \sin\gamma \cdot y_{2}+\cos\lambda \cdot y_{3}\\ \sin\lambda \cdot \cos\gamma \cdot z_{1}+\sin\lambda \cdot \sin\gamma \cdot z_{2}+\cos\lambda \cdot z_{3} \end{array}\right)$$

where $X_{L}={\left(\begin{array}{ccc} x_{1} & y_{1} & z_{1} \end{array}\right)}^{T}=T_{\max}^{S}/\left|T_{\max}^{S}\right|$, $Y_{L}={\left(\begin{array}{ccc} x_{2} & y_{2} & z_{2} \end{array}\right)}^{T}=T_{\min}^{S}/\left|T_{\min}^{S}\right|$, and $Z_{L}={\left(\begin{array}{ccc} x_{3} & y_{3} & z_{3} \end{array}\right)}^{T}=N/\left|N\right|$, are the representation of the coordinate axes of $CS_{L}$ in $CS_{W}$.

When the workpiece is fixed on the machine table, the $CS_{W}$ changes with the motion of the machine rotational axis, so T in $CS_{W}$ can be also represented as:

(3) $$T_{CS_{W}}=\left(\begin{array}{c} i\\ j\\ k \end{array}\right)=\left(\begin{array}{c} \cos C\cdot \sin B\\ \sin C\cdot \sin B\\ \cos B \end{array}\right)$$

Combining Equations (2) and (3), the relationship between the tool orientation and the rotational angle of the machine rotational axis can be represented as:

(4) $$\left(\begin{array}{c} \cos C\cdot \sin B\\ \sin C\cdot \sin B\\ \cos B \end{array}\right)=\left(\begin{array}{ccc} x_{1} & x_{2} & x_{3}\\ y_{1} & y_{2} & y_{3}\\ z_{1} & z_{2} & z_{3} \end{array}\right)\left(\begin{array}{c} \sin\lambda \cdot \cos\gamma \\ \sin\lambda \cdot \sin\gamma \\ \cos\lambda \end{array}\right)$$

**Figure 3:**
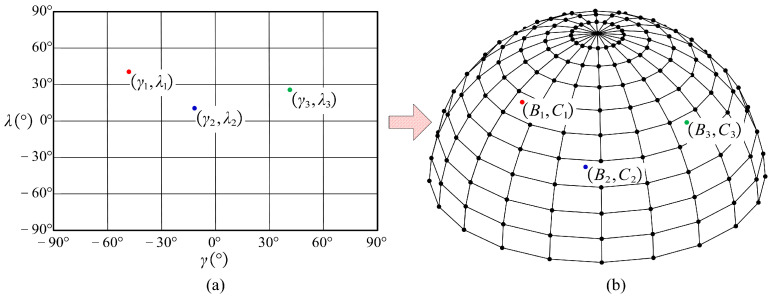
The relationship between the tool orientation and the angle of the machine rotational axis. (**a**) Representation of the tool orientation in the local coordinate system; (**b**) The angle of the machine rotational axis corresponding to the tool orientation.

Thus, based on the local inclination and local tilt angles at any *CC* point, the rotational angle of the machine rotational axis can be obtained by Equation (4), as shown in Figure 3.

### 2.2. Feasible Area of Tool Orientation and Tool Orientation Adjustment Method

In multi-axis CNC machining, the linkages between the axes are flexible, making interference problems easier to occur between the tool and the workpiece. Interference problems can affect the machining quality of the workpiece, scrap the part, seriously damage the machine spindle, or even cause problems such as machine scrapping. Thus, in five-axis CNC machining, it is necessary to avoid interference between the tool and the machined surface [61]. To obtain an interference-free tool path, it is necessary to calculate each *CC* point and guarantee that the tool orientation meets the basic requirements of tool path planning [25].

**Figure 4:**
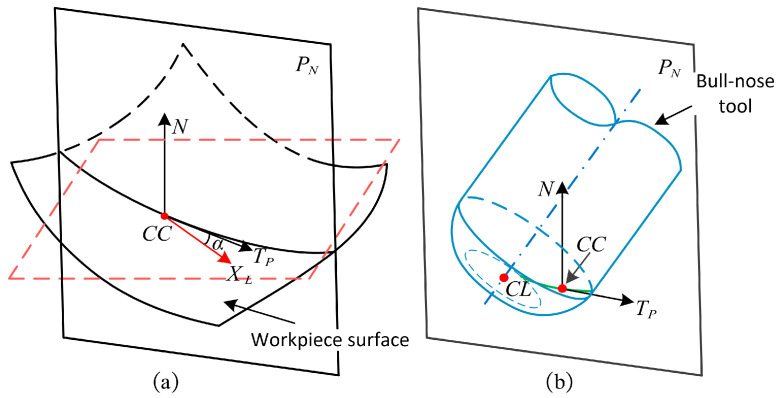
The configuration of the normal evaluation plane $P_{N}$. (**a**) The intersection between the normal evaluation plane and the workpiece surface; (**b**) The intersection between the normal evaluation plane and the tool.

As shown in Figure 4, the plane passing through the *CC* point and parallel to N is referred to as the normal evaluation plane and is denoted as $P_{N}$, and α is the angle between $P_{N}$ and $X_{L}$. On $P_{N}$, the difference between the curvature $\kappa_{N}^{C}\left(\alpha \right)$ and $\kappa_{N}^{S}\left(\alpha \right)$ is referred to as the evaluation curvature $\kappa_{N}\left(\alpha \right)$:

(5) $$\kappa_{N}\left(\alpha \right)=\kappa_{N}^{C}\left(\alpha \right)-\kappa_{N}^{S}\left(\alpha \right)$$

where $\kappa_{N}^{C}\left(\alpha \right)$ and $\kappa_{N}^{S}\left(\alpha \right)$ are respectively the normal curvature of the tool and workpiece surface on $P_{N}$. According to Euler’s criterion, $\kappa_{N}^{S}\left(\alpha \right)$ can be calculated as:

(6) $$\kappa_{N}^{S}\left(\alpha \right)=\kappa_{\max}^{S}\cdot \cos^{2}\left(\alpha \right)+\kappa_{\min}^{S}\cdot \sin^{2}\left(\alpha \right)$$

where $\kappa_{\max}^{S}$ and $\kappa_{\min}^{S}$ are the principal curvatures of the workpiece surface, respectively. Similarly, $\kappa_{N}^{C}\left(\alpha \right)$ can be calculated as:

(7) $$\kappa_{N}^{C}\left(\alpha \right)=\kappa_{\max}^{C}\cdot \cos^{2}\left(\alpha -\gamma \right)+\kappa_{\min}^{C}\cdot \sin^{2}\left(\alpha -\gamma \right)$$

where $\kappa_{\max}^{C}$ and $\kappa_{\min}^{C}$ are the principal curvatures of the tool, respectively. When the condition $\kappa_{N}\left(\alpha \right)\geq 0$ is met on normal evaluation planes, local interference does not occur at the *CC* point; when the condition $\kappa_{N}\left(\alpha \right)<0$ is satisfied at least on one normal evaluation plane, local interference occurs at the *CC* point.

Analyzing $\kappa_{N}\left(\alpha \right)$ of the normal evaluation plane $P_{N}$, and associating Equations (5) to (7), we have:

(8) $$\kappa_{N}\left(\alpha \right)=\frac{1}{2}\left(E+F\right)+\frac{1}{2}M\cdot \cos\left(2\alpha -\alpha \prime \right)$$

where $E=\kappa_{\max}^{C}\cdot \cos^{2}\gamma +\kappa_{\min}^{C}\cdot \sin^{2}\gamma -\kappa_{\max}^{S}$, $F=\kappa_{\max}^{C}\cdot \sin^{2}\gamma +\kappa_{\min}^{C}\cdot \cos^{2}\gamma -\kappa_{\min}^{S}$, $G=\sin2\gamma \cdot \left(\kappa_{\max}^{C}-\kappa_{\min}^{C}\right)$, $M=\sqrt{{\left(E-F\right)}^{2}+G^{2}}$, $\alpha^{\prime }=\arctan\left(\frac{E-F}{G}\right)$. From Equation (8), the minimum value of $\kappa_{N}\left(\alpha \right)$ is referred to as the critical evaluation curvature and is denoted as $\kappa_{Critical}$:

(9) $$\kappa_{Critical}=\frac{1}{2}\left(E+F-M\right)$$

**Figure 5:**
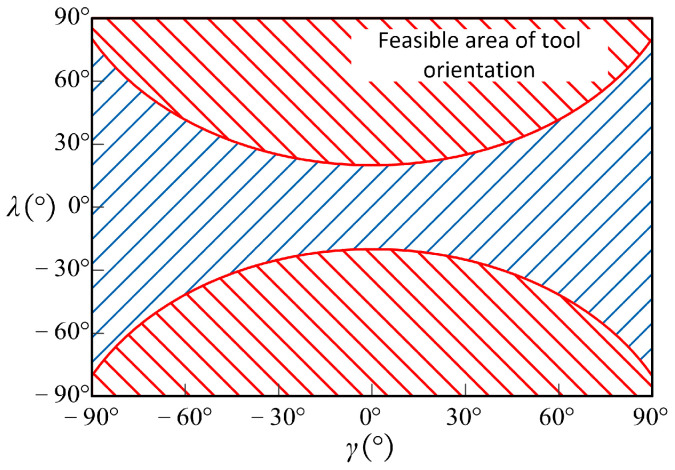
The feasible area of tool orientation.

Since $\kappa_{Critical}$ is the minimum of the evaluation curvature of all normal evaluation planes, the tool orientation is referred to as the feasible tool orientation if $\kappa_{Critical}\geq 0$ and no local interference occurs. The set of all feasible tool orientations is referred to as the feasible area of tool orientation, as shown in Figure 5. When the tool orientation is in the feasible area, the following equation holds:

(10) $$\sin\left|\lambda \right|\geq \frac{\left\{\kappa_{\max}^{S}\cdot \kappa_{\min}^{S}-\frac{1}{r}\left[\left(\kappa_{\max}^{S}-\kappa_{\min}^{S}\right)\cdot \sin^{2}\gamma +\kappa_{\min}^{S}\right]\right\}\cdot \left(R-r\right)}{\left(\kappa_{\max}^{S}+\kappa_{\min}^{S}\right)-\frac{1}{r}-r\cdot \kappa_{\max}^{S}\cdot \kappa_{\min}^{S}}$$

When machining the *CC* point, if local interference occurs, it is necessary to adjust the tool orientation to eliminate the interference and make it fall within the feasible area. The feasible area of tool orientation contains numerous options, and the process of adjusting the tool orientation is described below.

The initial tool orientation $\left(\gamma_{init},\lambda_{init}\right)$ is outside the feasible area when machining the *CC* point. For the convenience of calculation, take $\gamma_{r}=r\cdot \Delta \gamma ,r=1,2,\ldots ,180/\Delta \gamma -1$ and substitute it into Equation (10) to obtain the tool orientation corresponding to the boundary of the feasible area at this *CC* point, which is referred to as the potential interference-free tool orientation and denoted as $\left(\gamma_{r},\lambda_{r}\right)$. The distance $\Delta d_{r}$ between each $\left(\gamma_{r},\lambda_{r}\right)$ and the $\left(\gamma_{init},\lambda_{init}\right)$ is:

(11) $$\Delta d_{r}=\sqrt{{\left(\gamma_{r}-\gamma_{init}\right)}^{2}+{\left(\lambda_{r}-\lambda_{init}\right)}^{2}}.$$

**Figure 6:**
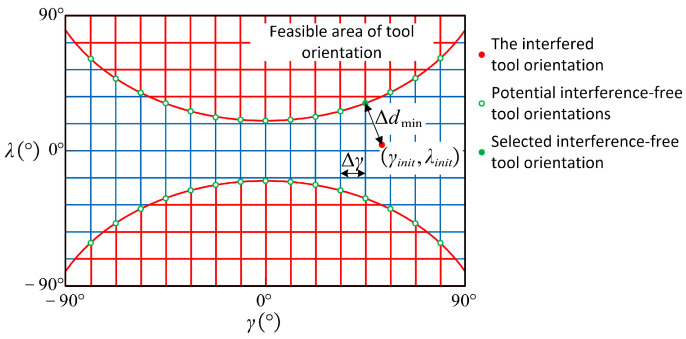
The schematic diagram of the distance between the initial and potential tool orientations.

The potential tool orientation with the shortest distance $\Delta d_{\min}$ from the initial tool orientation is the selected tool orientation, as shown in Figure 6.

## 3. The Machining Strip Width Calculation and Tool Orientation Adjustment Method on the Macro-Scale

### 3.1. Calculation Method of Machining Strip Width with a Bull-Nose Tool

**Figure 7:**
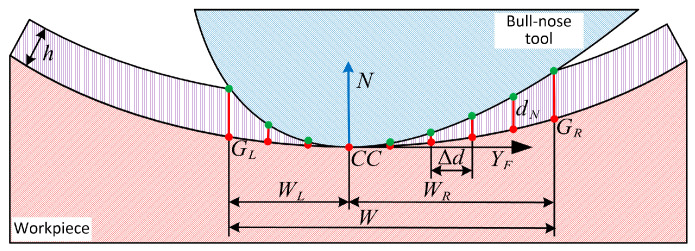
The schematic diagram for calculating the machining strip width.

The machining strip width can be obtained by intersecting the scallop surface with the tool in the plane $Y_{F}O_{F}Z_{F}$ perpendicular to the feed direction. Assuming a specified tolerance h for the workpiece surface, the scallop surface $S^{\prime }$ is obtained by offsetting the surface by h in the direction away from the workpiece. The tool orientation affects the projection of the bull-nose tool on $Y_{F}O_{F}Z_{F}$. Meanwhile, due to the special geometry of the bull-nose tool, it is impossible to approximate the tool’s geometry in this plane by a close circle at the *CC* point. As illustrated in Figure 7, in the plane $Y_{F}O_{F}Z_{F}$, a series of sampling points are planned on the workpiece surface along $Y_{F}$. The spacing between adjacent sampling points is denoted as Δd. The distance between each sampling point along N and the tool is referred to as the normal distance and is denoted as $d_{N}$. The sampling points are planned on both sides of the *CC* point, and the normal distance corresponding to each sampling point can be calculated. The sampling points corresponding to Δd=h are the boundaries of the machining strip width, denoted as $G_{L}$ and $G_{R}$, respectively. The length of the projection of $\vec{G_{L}G_{R}}$ on $Y_{F}$ is referred to as the machining strip width W.

(12) $$W=\left|\vec{G_{L}G_{R}}\cdot Y_{F}\right|.$$

Observing in the negative direction of $Y_{F}$, the machining strip width on the left side of the *CC* point is denoted as $W_{L}$, and the machining strip width on the right side is denoted as $W_{R}$.

(13) $$W=W_{L}+W_{R}$$

**Figure 8:**
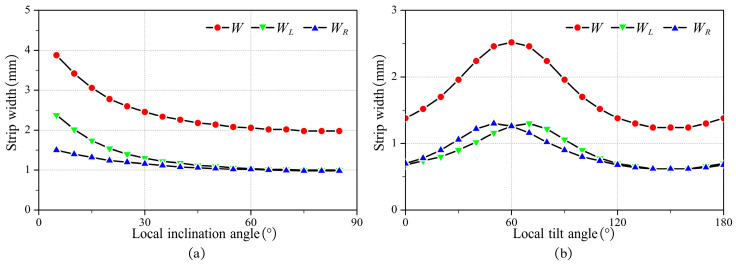
The effect of tool orientation on machining strip width (**a**) $\gamma =75^{\circ }$, $\sigma =60^{\circ }$ (**b**) $\gamma =30^{\circ }$, $\sigma =60^{\circ }$.

The tool orientation affects the machining strip width. With the above machining strip width calculation method, the effect of tool orientation on it can be easily calculated. Figure 8 presents the effect of local inclination and local tilt angles on W, $W_{L}$, and $W_{R}$ respectively.

As illustrated in Figure 8a, without local interference, the machining strip width decreases as the local inclination angle increases. As presented in Figure 8b, without local interference, the machining strip width increases and then decreases as the local tilt angle gradually increases from 0 to 180°. The maximum W appears when γ=σ, i.e., W reaches the maximum when the feed direction of the tool is the same as $T_{\max}^{S}$. Additionally, due to the special geometric properties of the bull-nose tool, $W_{L}$ and $W_{R}$ are equal when γ=σ.

### 3.2. Tool Orientation Adjustment Method to Keep Machining Strip Width Uniform

From a geometric perspective, a larger machining strip width corresponds to a higher material removal rate. However, when the machined surface is a freeform surface, the curvature of each *CC* point differs. Therefore, even if the tool orientation remains unchanged, it still leads to changes in W. In five-axis CNC milling, significant fluctuations in cutting force arise from the rapid changes in strip width, which consequently reduce tool life and machining quality [38]. Therefore, to avoid the problem of frequent changes in machining strip width that may cause a decrease in workpiece quality, it is necessary to keep the machining strip width as uniform as possible in the machining process.

**Figure 9:**
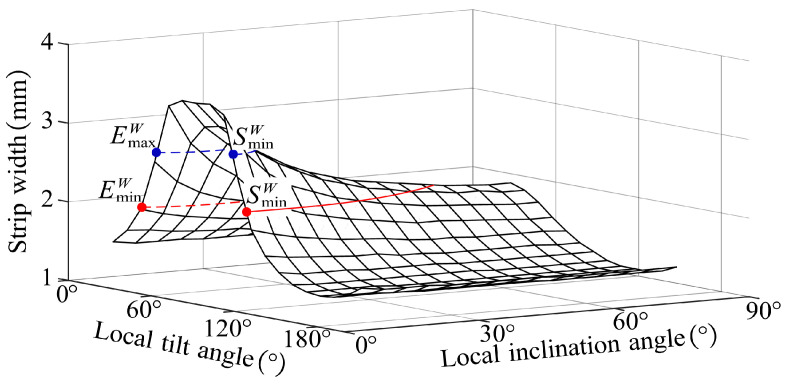
The relationship between the expected machining strip width and tool orientation.

To maintain a uniform machining strip width, it is necessary to adjust the tool orientation at each *CC* point. By changing the tool orientation, it is expected that the machining strip width at any *CC* point will converge to the expected machining strip width $W_{e}\in \left[W_{\min},W_{\max}\right]$.

(14) $$\left\{ \begin{array}{l} W_{\min}=2\sqrt{R_{eff}^{2}-{\left(R_{eff}^{}-h\right)}^{2}}-\frac{R}{10}\\ W_{\max}=2\sqrt{R_{eff}^{2}-{\left(R_{eff}^{}-h\right)}^{2}}+\frac{R}{10} \end{array}\right.$$

where $R_{eff}$ is the curvature radius of the bull-nose tool at the *CC* point on the plane $Y_{F}O_{F}Z_{F}$. In Figure 9, when the tool orientation is within the range of curves $S_{\max}^{W}E_{\max}^{W}$ and $S_{\min}^{W}E_{\min}^{W}$, the machining strip width falls within $W_{e}$. It is better to adjust the local inclination angle instead of the local tilt angle to change W, and this is because the change in the local inclination angle has a more significant influence on W than the local tilt angle. Besides, adjusting the local tilt angle results in a larger difference between $W_{L}$ and $W_{R}$. To maintain balance in material removal on both sides of the *CC* point during processing, it is preferable to select the tool orientation of γ=σ.

## 4. The Roughness Calculation and Tool Orientation Adjustment Method on the Micro-Scale

### 4.1. 2D Profile Simulation Model and Roughness Calculation Method

**Figure 10:**
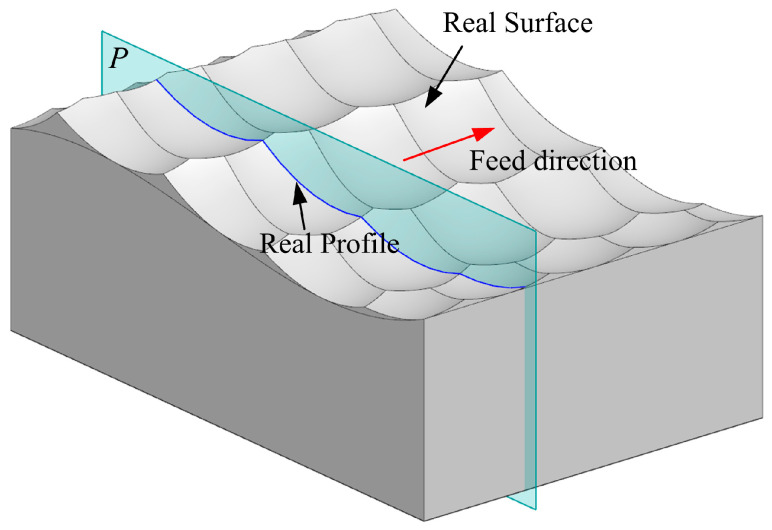
The real surface and profile of a free-form workpiece.

After using a bull-nose tool for five-axis CNC machining, a specific surface topography will be exhibited on the surface of the workpiece. From a geometric perspective, the surface topography is related to the step distance d and the feed per tooth $f_{z}$. Since $f_{z}$ is very small, it can be considered the scallops left by the tool motion as a part of the tool’s toric surface, as illustrated in Figure 10. The surface topography can be approximated as a series of scallops, with each scallop being determined by the *CC* point, principal direction, and principal curvature. The expression of the approximate surface of the scallop in the local coordinate system $\left\{CC,T_{\max}^{C},T_{\min}^{C},N\right\}$ is:

(15) $$z=\frac{1}{2}\left(\kappa_{\max}^{C}\cdot x^{2}+\kappa_{\min}^{C}\cdot y^{2}\right)+o\left(x^{2}+y^{2}\right)$$

The profile method is an important method for evaluating surface topography and has been widely used. As an essential characteristic of surface topography, roughness Ra is the difference between the real profile and the nominal profile within the evaluation length L. Generally, roughness is evaluated on a plane P that is perpendicular to the feed direction [62]. The evaluation length is determined by the standard, and the real profile refers to the intersection of the plane P with the real surface. The nominal profile is the curve obtained by the intersection of the plane P with the workpiece surface, and it is a curve on the theoretical surface $\overset{⌢}{p_{S}p_{E}}$.

**Figure 11:**
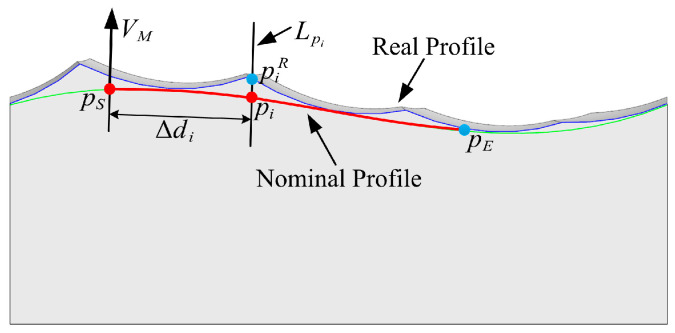
The difference between the real profile and the nominal profile.

Roughness Ra also refers to the difference between the real and nominal profiles. The line $L_{p_{i}}$ passes through point $p_{i}$ on the nominal profile, and it is parallel to the measurement direction $V_{M}$. The intersection points of $L_{p_{i}}$ with real profile is $p_{i}^{R}$ corresponds to $p_{i}$, as shown in Figure 11.

**Figure 12:**
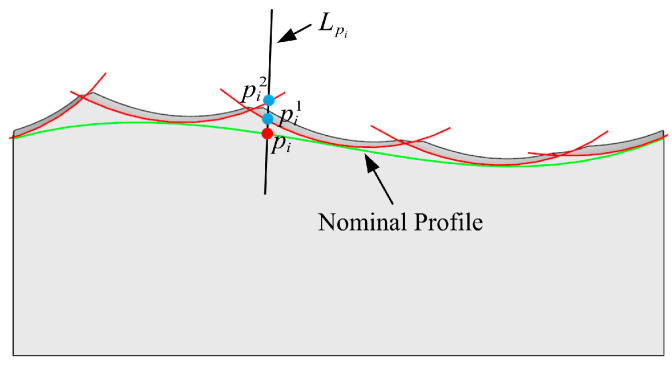
The intersection between $L_{p_{i}}$ and the real profile.

The line $L_{p_{i}}$ intersects with several scallops, and the distance between the point $p_{i}^{m}$ and the point $p_{i}$ is denoted as $l_{i}^{m}$, where m is the index of the scallop. The point corresponding to the minimum $l_{i}^{m}$ is the intersection point $p_{i}^{R}$ between $L_{p_{i}}$ and the real profile, as illustrated in Figure 12.

The steps to calculate the 2D real profile are as follows:

- Input the workpiece surface, tool parameters, and CL data.
- Calculate the discrete point $p_{i}$ on the nominal profile and obtain $L_{p_{i}}$ according to $p_{i}$ and the measurement direction $V_{M}$.
- Calculate the intersection point $p_{i}^{m}$ between $L_{p_{i}}$ and the scallop whose index is m. Then, calculate the distance $l_{i}^{m}$.
- Calculate the intersection point between $L_{p_{i}}$ and the next scallop and update the minimum distance $l_{i,\min}^{}$.
- Repeat steps (c) and (d) for each scallop to obtain the minimum distance $l_{i,\min}^{}$ and $p_{i}^{R}$.
- Repeat steps (b) to (e), for each discrete point $p_{i}$ to obtain the real profile.

After the real and nominal profiles are obtained, the roughness of the workpiece can be calculated based on the difference between the two. Ra is the average difference between the real profile and the mean profile in the evaluation length L, and it can be expressed as:

(16) $$Ra=\frac{\left(\left|Z_{1}\right|+\left|Z_{2}\right|+\ldots +\left|Z_{M}\right|\right)}{M}$$

**Figure 13:**
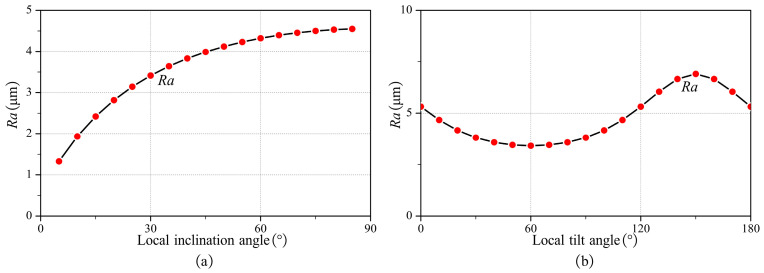
The influence of local inclination angle and local tilt angle on roughness. (**a**) $\gamma =60^{\circ }$, $\sigma =60^{\circ }$, d=0.8 mm, and $f_{z}=0.1\;\mathrm{mm}/\mathrm{tooth}$ (**b**) $\lambda =30^{\circ }$, $\sigma =60^{\circ }$, d=0.8 mm, and $f_{z}=0.1\;\mathrm{mm}/\mathrm{tooth}$.

The tool orientation significantly affects the roughness Ra. Based on the above calculation method of Ra, the influence of the tool orientation on the roughness Ra can be easily calculated. Figure 13 shows the influence of local inclination angle and local tilt angle on roughness. As illustrated in Figure 13a, without local interference, the roughness increases with the local inclination angle. As shown in Figure 13b, without local interference, as the local tilt angle gradually increases from 0 to 180°, the roughness first decreases and then increases. When γ=σ, the roughness Ra reaches its minimum.

### 4.2. Tool Orientation Adjustment Method to Meet Roughness Requirement

**Figure 14:**
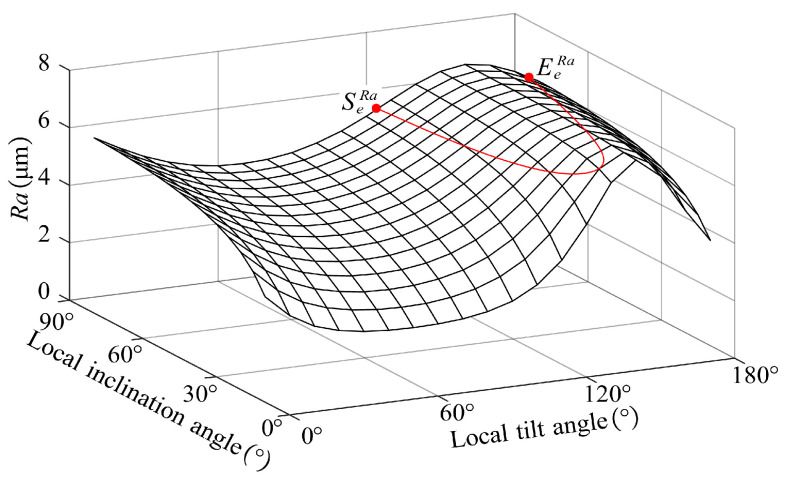
The relationship between roughness requirement and tool orientation.

In the machining process, the roughness Ra must meet the design requirements. By adjusting the tool orientation, it is expected that the roughness Ra at any *CC* point is smaller than the design requirements. As illustrated in Figure 14, if there is a curve $S_{e}^{Ra}E_{e}^{Ra}$, selecting any tool orientation below the curve causes the roughness Ra to be smaller than the design requirements. Compared with the local tilt angle, the effect of adjusting the local inclination angle on the roughness is more significant. Meanwhile, as the local inclination angle decreases, the roughness also gradually decreases. Therefore, when the roughness of the workpiece cannot meet the design requirements, the method of reducing the local inclination angle can be adopted to control the roughness.

## 5. Multi-Scale Tool Orientation Generation Method

The multi-scale tool orientation generation method aims to generate tool orientations that satisfy both the macro-scale machining strip width constraint and the micro-scale roughness constraint.

### 5.1. Method for Generating a Tool Orientation with a Smooth Rotational Axis

The tool orientation is nonlinear during the conversion between the workpiece coordinate system $CS_{W}$ and the machine coordinate system $CS_{M}$, so a smooth tool orientation in the $CS_{W}$ alone cannot ensure equally smooth rotation in a five-axis machine [56]. Poor tool orientation smoothing may cause discontinuous machine motion, which can lead to problems such as increased machining time and damage to surface quality. Therefore, when planning the tool orientation, the variation of the machine’s rotational axis should be considered to ensure smooth motion of the machine’s rotational axis.

The *CC* path is a continuous curve on the surface of the workpiece, as denoted as s(u). The angle of the rotational axis corresponding to the *CC* point on the *CC* path is denoted as $\beta^{*}$, where ∗=B,C. In this paper, the distribution of the rotational axis is expressed in the form of B-spline in the following form:

(17) $$\beta^{*}\left(u\right)=\sum_{j=0}^{n}N_{j,3}\left(u\right)\cdot Q_{j}^{*},$$

where $N_{j,3}\left(u\right)$ represents the cubic B-sample basis function, $Q_{j}^{*}$ represents the control coefficient, and U represents the knot vector.

The angular velocity of the rotational axis is an important parameter. To constrain the angular velocity to achieve a smooth rotational axis and to facilitate the calculation, the angular velocity is approximated by using the numerical differentiation method as follows:

(18) $$\omega_{i}^{*}=\frac{\beta^{*}\left(u_{i+1}\right)-\beta^{*}\left(u_{i}\right)}{\Delta t_{i}}$$

where $\Delta t_{i}=\left|\vec{s\left(u_{i}\right)s\left(u_{i+1}\right)}\right|/f$, and f is the feedrate during machining. To ensure smooth motion of the rotational axis, the angular variation should be as small as possible at adjacent *CC* points. That is, the rotational axis is considered smooth if the sum of the squares of the angular velocities at all *CC* points is minimized, i.e.,

(19) $$\Omega =\sum_{i=1}^{n-1}{\left(\omega_{i}^{*}\right)}^{2}$$

The least-square (LS) objective function shown in (20) can be obtained by substituting Equations (17) and (18) into Equation (19), which minimizes the angular velocity of the rotation axis by adjusting the control coefficient $Q_{j}^{*}$.

(20) $$\Omega =\sum_{i=1}^{n-1}{\left[\sum_{j=0}^{n}\left(\frac{N_{j,3}\left(u_{i+1}\right)-N_{j,3}\left(u_{i}\right)}{\Delta t_{i}}\right)Q_{j}^{*}\right]}^{2}.$$

The necessary condition for the minimum of the LS objective function is $\frac{\partial \Omega }{\partial Q_{k}^{*}}=0$, and k is the index of the control coefficient.

(21) $$\frac{\partial \Omega }{\partial Q_{k}^{*}}=\sum_{i=1}^{n-1}\left\{\left[2\times \sum_{j=0}^{n}\left(\frac{N_{j,3}\left(u_{i+1}\right)-N_{j,3}\left(u_{i}\right)}{\Delta t_{i}}\right)Q_{j}^{*}\right]\times \frac{N_{k,3}\left(u_{i+1}\right)-N_{k,3}\left(u_{i}\right)}{\Delta t_{i}}\right\}=0$$

At the start and end of each tool path, the tool orientation is given. Meanwhile, the first and last control coefficient $Q_{0}^{*}$ and $Q_{n}^{*}$ are the same as the rotation angles $\beta_{}^{*}\left(0\right)$ and $\beta_{}^{*}\left(1\right)$ of the rotational axis. Substituting $Q_{0}^{*}=\beta_{}^{*}\left(0\right)$ and $Q_{n}^{*}=\beta_{}^{*}\left(1\right)$ into Equation (21), we have:

(22) $$\begin{array}{l} \sum_{j=1}^{n-1}\left\{\left[\sum_{i=1}^{n-1}\left(\frac{N_{k,3}\left(u_{i+1}\right)-N_{k,3}\left(u_{i}\right)}{\Delta t_{i}}\right)\left(\frac{N_{j,3}\left(u_{i+1}\right)-N_{j,3}\left(u_{i}\right)}{\Delta t_{i}}\right)\right]Q_{j}^{*}\right\}=\\ -\sum_{i=1}^{n-1}\left\{\left[\left(\frac{N_{0,3}\left(u_{i+1}\right)-N_{0,3}\left(u_{i}\right)}{\Delta t_{i}}\right)Q_{0}^{*}+\left(\frac{N_{n,3}\left(u_{i+1}\right)-N_{n,3}\left(u_{i}\right)}{\Delta t_{i}}\right)Q_{n}^{*}\right]\cdot \frac{N_{k,3}\left(u_{i+1}\right)-N_{k,3}\left(u_{i}\right)}{\Delta t_{i}}\right\} \end{array}$$

Bredies, other constraints need to be added to the above set of equations. For instance, if some *CC* points are found to have local interference or the machining strip width does not meet the expectation, their corresponding tool orientations should be corrected according to the adjustment method in the previous section and set to fixed values. Suppose there are p *CC* points and their fixed tool orientations are $\beta_{}^{*}\left(u_{p}\right)$. the curve of the rotational axis angle needs to be constrained by these tool orientations, i.e.,

(23) $$\beta_{}^{*}\left(u_{p}\right)=\sum_{j=0}^{n}N_{j,3}\left(u_{p}\right)\cdot Q_{j}^{*}.$$

Substituting $Q_{0}^{*}=\beta_{}^{*}\left(0\right)$ and $Q_{n}^{*}=\beta_{}^{*}\left(1\right)$ into Equation (23) yields:

(24) $$\sum_{j=1}^{n-1}N_{j,3}\left(u_{p}\right)Q_{j}^{*}=\beta_{}^{*}\left(u_{p}\right)-Q_{0}^{*}N_{0,3}\left(u_{p}\right)-Q_{n}^{*}N_{n,3}\left(u_{p}\right)$$

Combining Equations (22) and (24), a set of linear equations can be obtained with the control coefficient $Q_{j}^{*}$, j=1,2,…,n−1 as the unknown:

(25) $$NQ^{*}=R^{*},$$

where $Q^{*}={\left[\begin{array}{cccc} Q_{1}^{*} & Q_{2}^{*} & \cdots & Q_{n-1}^{*} \end{array}\right]}^{T}$, N is a (n+p−1)×(n−1) coefficient matrix, and $R^{*}$ is a (n+p−1)×1 vector. The control coefficient can be solved by the following equation:

(26) $$Q^{*}={\left(N^{T}N\right)}^{-1}N^{T}R^{*}.$$

### 5.2. Multi-Scale Tool Orientation Generation Method

**Figure 15:**
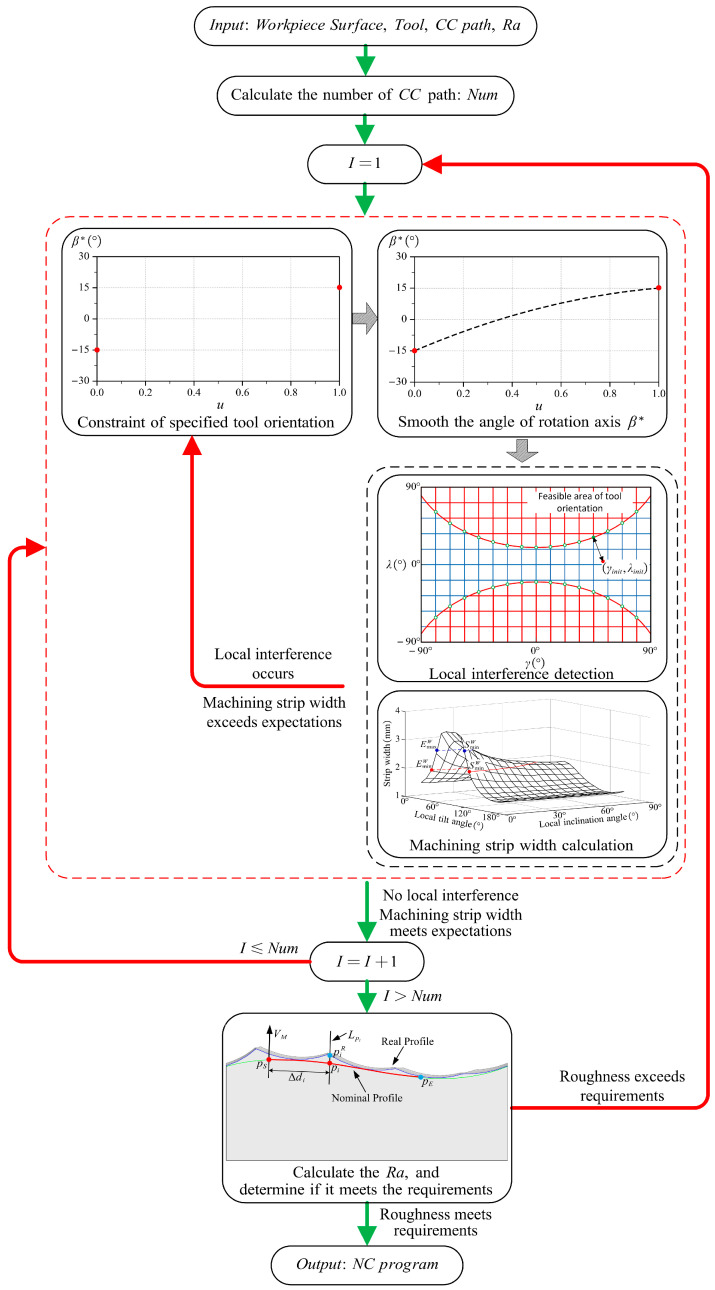
Flow of the multi-scale tool orientation generation method.

The implementation of the multi-scale tool orientation generation method proposed in this paper is shown in Figure 15. The input includes the workpiece surface, tool, *CC* path, and roughness requirements, and the output is the NC program (including CL and tool orientation) that meets the roughness requirements on the micro-scale and has uniform machining strip widths on the macro-scale.

Specifically, the generation method requires iterative calculation, which mainly includes the following four steps:

Before planning the tool orientation, some preparation work needs to be performed: first, find the evaluation length Ra in the standard according to the roughness requirement, and according to this, calculate the number of *CC* paths to be planned Num, and then the iterative calculation of the tool orientation for the above *CC* paths can be conducted.

Step 1: Specify the constraints for the tool orientation.

Step 2: According to Equation (26), calculate the smooth angle of the rotational axis $\beta^{*}$.

Step 3: According to the formula in Section 2.2, calculate the tool orientation (λ,γ) corresponding to each *CC* point in $CS_{L}$ and judge whether there is local interference or the machining strip width exceeds the expectation according to the description in Section 2 and Section 3. If these problems occur, adjust the tool orientation and add the corresponding tool orientations at these *CC* points to the constraints, and then execute steps 2 and 3 again until the tool orientations that satisfy the expectation of no local interference and machining strip width are generated.

When the planning of the tool orientation of one *CC* path is completed, the calculation of the next *CC* path is conducted until the tool orientations of Num *CC* paths are all planned.

Step 4: According to the roughness calculation method in Section 4, the roughness of the above Num *CC* paths is calculated to determine whether the roughness meets the design requirements. If the roughness does not meet the requirement, adjust the tool orientation according to Section 4.2 and add the tool orientation corresponding to these *CC* points to the tool orientation constraint of the *CC* paths, and then execute steps (1) to (3) again until the tool orientations that meet the roughness requirements are generated.

The multi-scale tool orientation generation method proposed in this paper can ensure that no local interference occurs at any *CC* point. Meanwhile, it can ensure that the cutting bandwidth at the machining strip widths on the macro-scale is uniform and the roughness requirements on the micro-scale are met. Additionally, this method can ensure the smoothness of the rotational axis. During the machining process, kinematic singularities occur when the tool orientation passes through singular points, causing the rotation axis to move over a large range in a short period of time [63]. Consequently, when the angle of the rotational axis experiences abrupt changes at adjacent CC points, singularities appear, and the constraint of the tool orientation must be reassigned. Furthermore, since the objective of this study is to generate tool orientations that satisfy both macro-scale and micro-scale requirements, only the smoothing of the rotational axis is considered. To the best of our knowledge, maintaining continuous and smooth velocity for the rotational axis during the machining process also ensures continuous and smooth velocity, acceleration, and jerk for the machine tool driver. However, this aspect warrants further investigation.

## 6. Experiment

**Figure 16:**
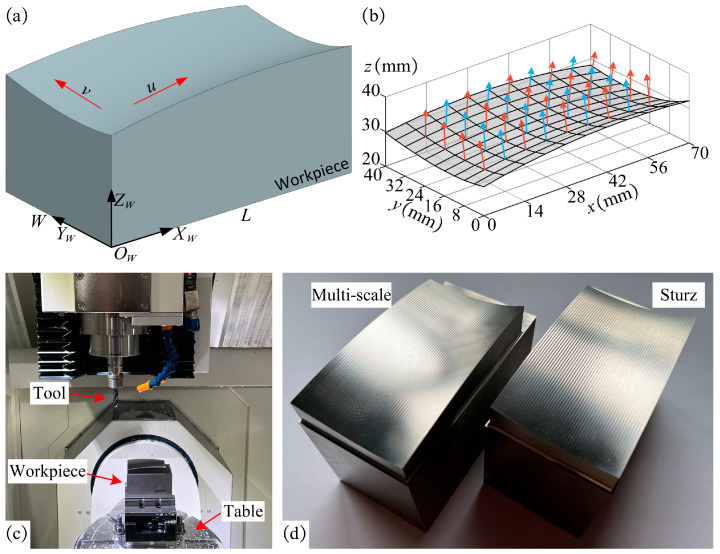
(**a**) Model of the workpiece with a free-form surface; (**b**) normal vectors on the free-form surface; (**c**) configuration of the experiment; (**d**) free-form surface being machined.

To verify the effectiveness of the proposed multi-scale tool orientation generation method, it was applied to the machining of a free-form surface. The iso-parametric method is employed to generate *CC* paths with Δv=0.02, and the interval between the adjusted *CC* points is, Δu=0.01, as shown in Figure 16a. Besides, the Sturz [64] method and the multi-scale method are adopted to plan the tool orientation, respectively. Specifically, the Sturz method has an inclination angle of 20° and a tilt angle of 0°. The tool orientation of the method proposed in this paper is consistent with the Sturz method at the starting and end points, both of which have an inclination angle of 20° and a tilt angle of 0°, and then the tool orientations at other *CC* points are planned. The workpiece roughness requirement is Ra=1.6 μm, and according to the standard, the evaluation length L=4 mm. According to Equation (14), the range of expected machining strip width is $W_{e}\in \left[2.15\;\mathrm{mm},3.15\;\mathrm{mm}\right]$. Figure 16b illustrates the distribution of the normal vector on the free-form surface. During the machining of free-form surfaces, variations in the normal vector influence the tool orientation, resulting in continuous changes throughout the machining process. Therefore, the experimental validation involves real 5-axis operations.

In this experiment, a 5-axis CNC machine tool with a BC double turntable structure (JDGR200T, Beijing, Jingdiao, China) was employed to machine the workpiece. The material used in the experiment was 2Cr13. Meanwhile, a 4-tooth bull-nose tool with a radius R=5 mm and corner radius r=3 mm was used for finishing. The machining parameters used in the experiment are as follows: the spindle speed is 4800 r/min, and the feedrate is 800 mm/min. The workpiece is fixed on the turntable as shown in Figure 16c. After machining the workpiece with the above two methods, the machined workpiece was obtained, as shown in Figure 16d.

**Figure 17:**
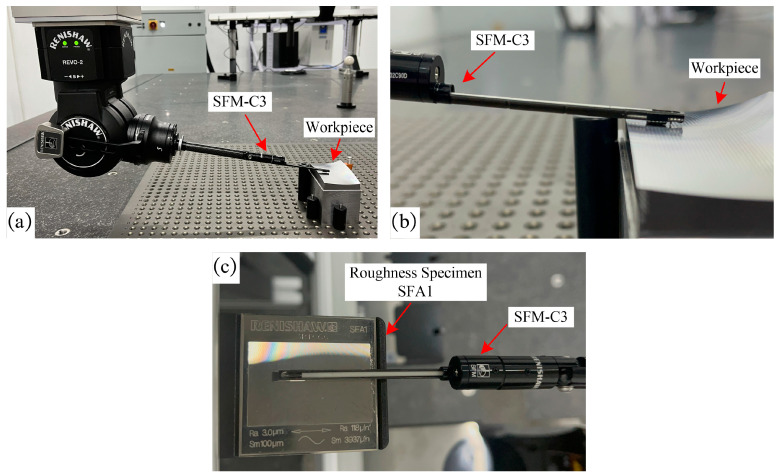
The Renishaw REVO^©^ 5-axis measurement system employed to measure the roughness. (**a**) The SFM-C3 surface finish module employed for roughness measurement; (**b**) SFM-C3 in contact with the workpiece during roughness measurement; (**c**) Calibration of the SFM-C3 surface finish module.

After machining, the roughness of the workpiece was measured at 90 different locations evenly distributed on the surface with the Renishaw REVO^©^ 5-axis measurement system. The experimental equipment comprised a Renishaw Agility 5-axis measurement system outfitted with an SFM-C3 surface finish module, as depicted in Figure 17a,b. The maximum permissible error of length measurement (MPE) in the 5-axis measurement system was 1.9 μm + L/350 μm, while maximum permissible limit of the repeatability range (MPL) was 1.3 μm. The SFM-C3’s accuracy (of nominal Ra) was ±10% + 35 nm. The SFM-C3 was calibrated prior to the measurement process with Renishaw Roughness Specimen SFA1, as demonstrated in Figure 17c. The measurements were conducted at a stable temperature of 20 °C, and the SFM-C3 operated at a speed of 1 mm/s during roughness measurements. The measurement results are shown in Table 1 and Table 2.

In the machining process, the minimum and maximum machining strip widths of the tool orientation generated by the Sturz method are, respectively $W_{\min}^{}=2.60\;\mathrm{mm}$ and $W_{\max}^{}=2.78\;\mathrm{mm}$, which fall within the expectation of machining strip width. It can be seen from Table 1 that there are 13 positions where the roughness does not meet the roughness requirements. Hence, the tool orientation obtained by the Sturz method can meet the machining strip width expectation but cannot achieve the required roughness.

In contrast, the minimum and maximum machining strip widths of the tool orientation generated by the multi-scale method are, respectively $W_{\min}^{}=2.60\;\mathrm{mm}$ and $W_{\max}^{}=2.84\;\mathrm{mm}$, which fall within the expectation of machining strip width too. From Table 2, it can be determined that the roughness at all positions meets the roughness requirements. Hence, the multi-scale method proposed in this paper can meet the machining strip width expectation, and at the same time, it ensures that the roughness meets the requirement.

Therefore, it can be concluded that the proposed multi-scale method can obtain the tool orientation that meets the requirements of the macro-scale and micro-scale.

## 7. Conclusions

In five-axis CNC machining, the machining quality of the workpiece is closely related to tool orientation. An appropriate tool orientation can help to avoid interference, keep the machining strip width uniform, control the roughness, etc. In this paper, by considering the influence of both machining strip width in the macro-scale and roughness in the micro-scale, a multi-scale tool orientation generation method is proposed for 5-axis CNC machining of bull-nose tools, which can ensure no interference and smoothness of the rotational axis during machining. Based on the conducted analyses and the obtained findings, the following conclusions can be drawn:

(1) The method is proposed to calculate the machining strip width of a free-form surface machined by a bull-nose tool, and the influence of the tool orientation on the machining strip width is analyzed. Meanwhile, to solve the problem that frequent changes in machining strip width reduce machining quality, a tool orientation adjustment method is proposed to maintain the machining strip width uniform in the machining process.
(2) The simulation model of the real profile of a free-form surface machined by a bull-nose tool is established, and the roughness is calculated based on this. Additionally, the influence of tool orientation on roughness is analyzed, and a tool orientation adjustment method is proposed to ensure that the roughness meets the design requirements.
(3) Based on the above models, a multi-scale tool orientation generation method is proposed that can ensure no local interference at any CC point and meet the requirements for machining strip width on a macro-scale and roughness on a micro-scale. Additionally, the proposed method can ensure the minimum angular velocity of the rotational axis.
(4) The experimental results indicate that the multi-scale method proposed in this paper is effective. It is experimentally verified that the tool orientation generated by the method can not only meet the requirement for machining strip widths but also ensure roughness, i.e., satisfy both macro-scale and micro-scale requirements.

This demonstrates that the method proposed in this paper has a wide prospect and potential for practical applications and can provide an effective solution for the industry.

## Figures and Tables

**Table 1:** Roughness measurement results using Sturz method.

*Ra* (μm)	1	2	3	4	5	6	7	8	9
1	1.220	1.252	1.381	1.158	1.286	1.295	1.573	1.081	1.292
2	1.330	1.292	1.312	1.275	1.249	1.243	1.201	1.194	1.838
3	1.548	1.427	1.363	1.440	2.087	1.265	1.098	1.461	0.905
4	1.582	1.615	1.467	1.563	1.343	1.389	1.146	1.230	1.215
5	1.599	1.412	1.456	1.485	1.436	1.326	1.331	1.302	1.183
6	1.477	1.509	1.553	1.523	1.299	1.404	1.345	1.111	1.898
7	1.510	1.505	1.605	1.609	1.365	1.465	1.340	1.258	1.118
8	1.513	2.027	1.710	1.482	1.557	1.684	1.326	1.395	1.316
9	1.607	1.510	1.539	1.631	1.496	1.511	1.421	1.289	1.186
10	1.580	1.472	1.720	1.464	1.580	1.985	1.368	1.491	1.311

**Table 2:** Roughness measurement results using multi-scale method.

*Ra* (μm)	1	2	3	4	5	6	7	8	9
1	0.869	0.839	0.910	0.828	0.921	0.800	0.791	0.745	0.719
2	0.968	0.876	1.025	1.090	1.024	0.908	0.909	0.766	0.791
3	1.070	1.060	1.148	1.125	1.159	1.141	1.029	0.926	0.852
4	1.198	1.128	1.175	1.100	1.271	1.108	1.032	0.980	0.852
5	1.183	1.199	1.145	1.159	1.168	1.072	1.063	0.982	0.828
6	1.151	1.099	1.111	1.097	1.106	1.050	1.132	0.945	0.868
7	1.128	1.137	1.158	1.050	1.085	1.158	1.096	0.942	0.973
8	1.019	1.041	1.169	1.153	1.196	1.168	1.083	0.901	0.869
9	1.005	1.159	1.103	1.059	1.110	1.009	1.029	0.954	0.856
10	1.113	1.085	1.034	1.080	1.117	1.019	0.956	0.908	0.835

## Data Availability

Not applicable.
